# Quantitative Accuracy and Precision in Multiplexed Single-Cell Proteomics

**DOI:** 10.1021/acs.analchem.1c04174

**Published:** 2021-12-30

**Authors:** Claudia Ctortecka, Karel Stejskal, Gabriela Krššáková, Sasha Mendjan, Karl Mechtler

**Affiliations:** † Research Institute of Molecular Pathology (IMP), Vienna BioCenter (VBC), Campus Vienna Biocenter 1, 1030 Vienna, Austria; ‡ Institute of Molecular Biotechnology of the Austrian Academy of Sciences (IMBA), Vienna BioCenter (VBC), Dr. Bohr-Gasse 3, 1030 Vienna, Austria; § The Gregor Mendel Institute of Molecular Plant Biology of the Austrian Academy of Sciences (GMI), Vienna BioCenter (VBC), Dr. Bohr-Gasse 3, 1030 Vienna, Austria

## Abstract

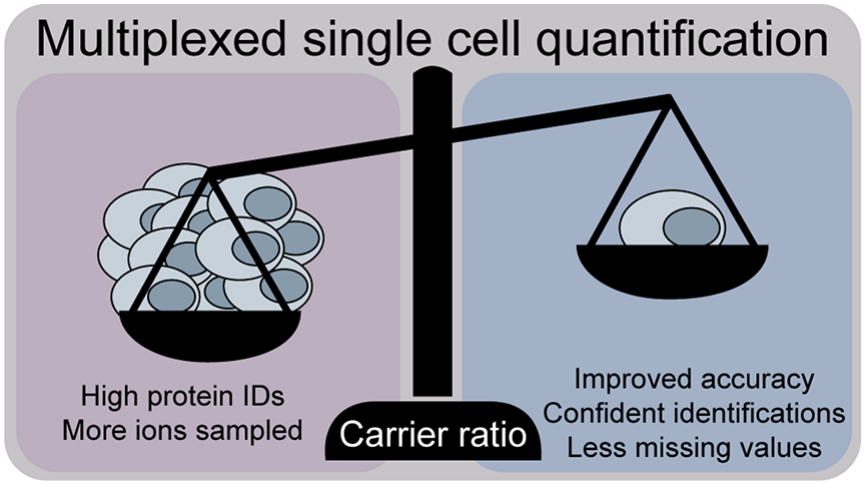

Single-cell proteomics workflows have considerably improved in sensitivity and reproducibility to characterize as-yet unknown biological phenomena. With the emergence of multiplexed single-cell proteomics, studies increasingly present single-cell measurements in conjunction with an abundant congruent carrier to improve the precursor selection and enhance identifications. While these extreme carrier spikes are often >100× more abundant than the investigated samples, the total ion current undoubtably increases but the quantitative accuracy possibly is affected. We here focus on narrowly titrated carrier spikes (i.e., <20×) and assess their elimination for a comparable sensitivity with superior accuracy. We find that subtle changes in the carrier ratio can severely impact the measurement variability and describe alternative multiplexing strategies to evaluate data quality. Lastly, we demonstrate elevated replicate overlap while preserving acquisition throughput at an improved quantitative accuracy with DIA-TMT and discuss optimized experimental designs for multiplexed proteomics of trace samples. This comprehensive benchmarking gives an overview of currently available techniques and guides the conceptualization of the optimal single-cell proteomics experiment.

## Introduction

Single-cell proteomics have been demonstrated as a viable complement to single-cell transcriptomics studies with striking sensitivity. Those single-cell analyses of presumed-homogeneous cell populations have attributed biological variability at both the transcriptome and proteome levels.^1,2^ Previously, most protein analyses with single-cell resolution have been antibody-based or were limited to large cells such as oocytes.^3,4^ More recent technological innovations now allow for the hypothesis-free proteome analysis of single mammalian cells.^5−7^ The first of such aimed at overcoming the limited sensitivity of available mass spectrometers through isobaric labeling.^3,8^ Isobaric labels use their identical mass with a different isobaric distribution, allowing the simultaneous analysis of multiple samples and their quantification upon fragmentation within one MS^*n*^ scan. SCoPE-MS (single-cell proteomics by mass spectrometry) combines tandem mass tag (TMT)-multiplexed single-cells with a 200 cell congruent carrier sample.^8^ The highly abundant carrier overcomes adsorptive losses before MS analysis, boosts the peptide signals during MS1 scans, and therefore increases the signal-to-noise ratio (S/N) of the peptide precursor and provides fragment ions for identification. Following the initial publication, such congruent carrier spikes were employed to improve the triggering of peptides of interest at varying ratios from 25× up to 500×.^7,9,10^

While an abundant carrier improves peptide identifications by increasing ion counts, selecting the appropriate acquisition parameters and carrier compositions are crucial to preserving the quantitative accuracy.^10−12^ Such imbalanced levels of multiplexed carrier samples with ratios of 200 or higher were demonstrated to possibly impact biological conclusions.^9,10,12,13^ The effects of extreme ratios on ion suppression,^14^ ratio compression,^15,16^ and quantification accuracy^9^ were previously described for standard and trace samples. The latter was addressed by increasing the number of ions sampled from each precursor,^11,17,18^ which was constrained by the injection time (IT) and the automatic gain control (AGC) target. However, increasing the total number of ions sampled per precursor capitalizes the ions originating from the carrier within imbalanced samples.^12,19^ Additionally, the thus-lengthened cycle times reduce the number of MS/MS scans within one analytical run, inflating missing data between replicates due to precursor stochasticity.^20^

Recently, Cheung and colleagues evaluated the “carrier proteome” effects for trace samples and proposed a maximum level of a congruent carrier (∼20×) for optimal ion statistics and quantification accuracy.^12^ They and others thoroughly discuss the need for appropriate MS acquisition parameters and S/N filtering when performing MS-based single-cell proteomics experiments.^11,12^ However, it remains unclear which levels of excess carrier provide the optimal balance between sensitivity and accuracy or whether even drastically reduced ratios impair quantitative precision. Therefore, we demonstrate the applicability and confirm the need for S/N filtering to improve data quality in various multiplexed experimental designs. Moreover, with alternative multiplexing or acquisition strategies, we discuss the impact of coisolation and precursor stochasticity in the analysis of trace samples. This study aims to outline the advantages of available experimental setups and compile critical aspects, including identification rates, measurement accuracy, acquisition variability, and missing quantitative data, in single-cell proteomics experiments.

## Methods

### Sample Preparation

Cells were pelleted, washed with phosphate-buffered saline (PBS), and stored at −80 °C until they were lysed using a methanol/chloroform/water solution (4:1:3), then sonicated and dried to completeness in a speed-vac concentrator. The dry protein pellets were resuspended in 8 M urea in 10 mM HCl. Prior to alkylation with iodoacetamide (40 mM, 30 min at room temperature (RT)), the samples were adjusted to 200 mM Tris/HCl pH 8.0 and reduced using dithiothreitol (50 mM, 37 °C, 30 min). The reduced and alkylated samples were diluted to a final concentration of 4 M urea in 100 mM Tris/HCl pH 8 and digested with LysC (Wako, enzyme/protein 1:100) for 3 h at 37 °C, if indicated. Tryptic samples were subsequently diluted to 2 M urea in 100 mM Tris/HCl pH 8 and digested overnight at 37 °C (Promega, enzyme/protein 1:100). Samples were adjusted to pH 2 using 10% trifluoroacetic acid (TFA), desalted using C18 solid-phase extraction cartridges (SPE, C18 Sep-pak, 200 mg Waters), and eluted with 40% acetonitrile (ACN) in 0.1% TFA. The SPE eluate volume was reduced using a vacuum centrifuge and labeled according to the manufacturer’s instructions. Briefly, samples were labeled in 100 mM TEAB and 10% ACN for 1 h at RT. The unreacted TMT reagent was quenched with 5% hydroxylamine/HCl for 20 min at RT and subsequently mixed corresponding to each sample pool.

### LC MS/MS Analysis

Samples were measured on a Orbitrap Exploris 480 Mass Spectrometer (Thermo Fisher Scientific) with a reverse-phase Dionex UltiMate 3000 high-performance liquid chromatography (HPLC) RSLCnano system coupled via a Nanospray Flex ion source equipped with FAIMS Pro (Thermo Fisher Scientific), which was operated at a constant compensation voltage of −50 V. Chromatographic separation was performed on a nanoEase M/Z Peptide BEH C18 column (130 Å, 1.7 μm, 75 μm × 150 mm, Waters, Germany) that developed a two-step solvent gradient ranging from 1.2% to 30% over 90 min and 30% to 48% ACN in 0.08% formic acid within 20 min at a flow rate of 250 nL/min.

SCoPE-MS and SCoPE2 acquisition strategies were performed as published with small adaptations. Briefly, full MS data were acquired in the range of 395–1800 or 450–1600 *m*/*z* at a resolution of 60000 for SCoPE-MS or SCoPE2 samples, respectively. The maximum AGC was set to 3 × 10^6^ and automatic IT. The top 20 or 7 multiply charged precursor ions (2–3 or 2–4) with a minimum intensity of 2 × 10^4^ were isolated for higher-energy collisional dissociation (HCD) MS/MS using a 1 or 0.7 with 0.3 Th offset isolation window, respectively. Precursors were accumulated until they either reached an AGC target of 1 × 10^5^ or a maximum IT of 250 or 300 ms, respectively. MS/MS data were generated with a normalized collision energy (%NCE) of 34 at a resolution of 60 000, with the first mass fixed to 110 *m*/*z*. Upon first fragmentation, precursor ions were dynamically excluded (dynEx) for 20 or 30 s, respectively.

Full MS data of multiplexed carrier experiments were acquired in a range of 375–1200 *m*/*z* with a maximum AGC target of 3 × 10^6^ and automatic an IT at 120 000 resolution. The top 10 multiply charged precursors (2–5) over a minimum intensity of 5 × 10^3^ were isolated using a 0.7 Th isolation window and acquired at a resolution of 60 000 at a fixed first mass of 110 *m*/*z* with a maximum AGC target of 1 × 10^5^ or an IT of 118 ms and dynEx of 120 s. TMT10-plex (TMT10) precursors were fragmented at an NCE of 34 and TMTpro at an NCE of 32.

TMT_zero_ experiments were performed similarly but precursors were selected using a “targeted mass-difference method” for a 3 s cycle time. For this, the δ-mass of 5.0105 or 10.0209 Da was used to select only precursors with a matching partner intensity to the most intense one, with a mass tolerance of 10 ppm. Targeted precursors were isolated with a 0.7 Th isolation window at an AGC target of 1 × 10^5^ for a maximum 250 ms IT. Selected precursors were acquired at a resolution of 45 000 with a dynEx of 100 s.

DIA-TMT experiments were performed in the range of 400–800 *m*/*z* at a resolution of 45 000. The AGC was set to 2 × 10^5^ with an automatic maximum IT and 5 Th isolation windows, including a 1 Th overlap with the first mass fixed to 120 *m*/*z*. This corresponds to 80 DIA windows with a cycle time of 6 s. TMT10 samples were fragmented with a stepped NCE of 30, 37.5, and 45 and TMTpro with 25, 30, and 40.

### Data Analysis

Reporter ion (RI) quantification was performed within the Proteome Discoverer environment (ver. 2.3.0.484) using the in-house-developed freely available PD node “IMP-Hyperplex” (pd-nodes.org) to extract the intensity and S/N values for all a RIs at a mass tolerance of 10 ppm. Quality control of raw data was performed using RawTools.^21^ Venn diagrams were generated using BioVenn.^22^

Peptide identification was performed using the standard parameters in SpectroMine 2.0 against the human reference proteome sequence database (UniProt; ver. 2018-11-26, accessed 2019-04). Briefly, we performed a specific tryptic search with a maximum of two missed cleavages, limiting peptides to 7–52 amino acids. We included carbamidomethylation on cysteins, TMT10 or TMTpro on lysine, all N-terms as fixed modifications, acetylation on protein N-terms, and a methionine oxidation variable. By default, SpectroMine performs ideal mass tolerance calculations at MS and MS/MS levels and mass calibration for each feature. Subsequently, identifications were filtered for 1% FDR on the PSM, peptide, and protein-group level for further processing.

For SCoPE-MS and SCoPE2 reanalysis, raw files were obtained from the following repositories and processed as indicated above (MSV000084660, MSV000083945, and MSV000082077).^7,8^

TMT spectral libraries were generated from the DDA files with the above indicated parameters using a customized script provided by Oliver Bernhard from Biognosys (available on GitHub as ctorteckac/DIA-TMT).^23^ Libraries were searched with Spectronaut by performing mass tolerance calculations and spectra matching based on extensive mass calibration. The most intense peak within the previously defined mass tolerance was then selected and matched with a minimum of three matching fragment ions per MS/MS scan. RT alignments are based on iRT Reference Strategy with minimum *R*^2^ = 0.8. “Mutated” decoys with scrambled sequences were filtered for 1% FDR on precursor and protein levels.

## Results and Discussion

**Figure 1:**
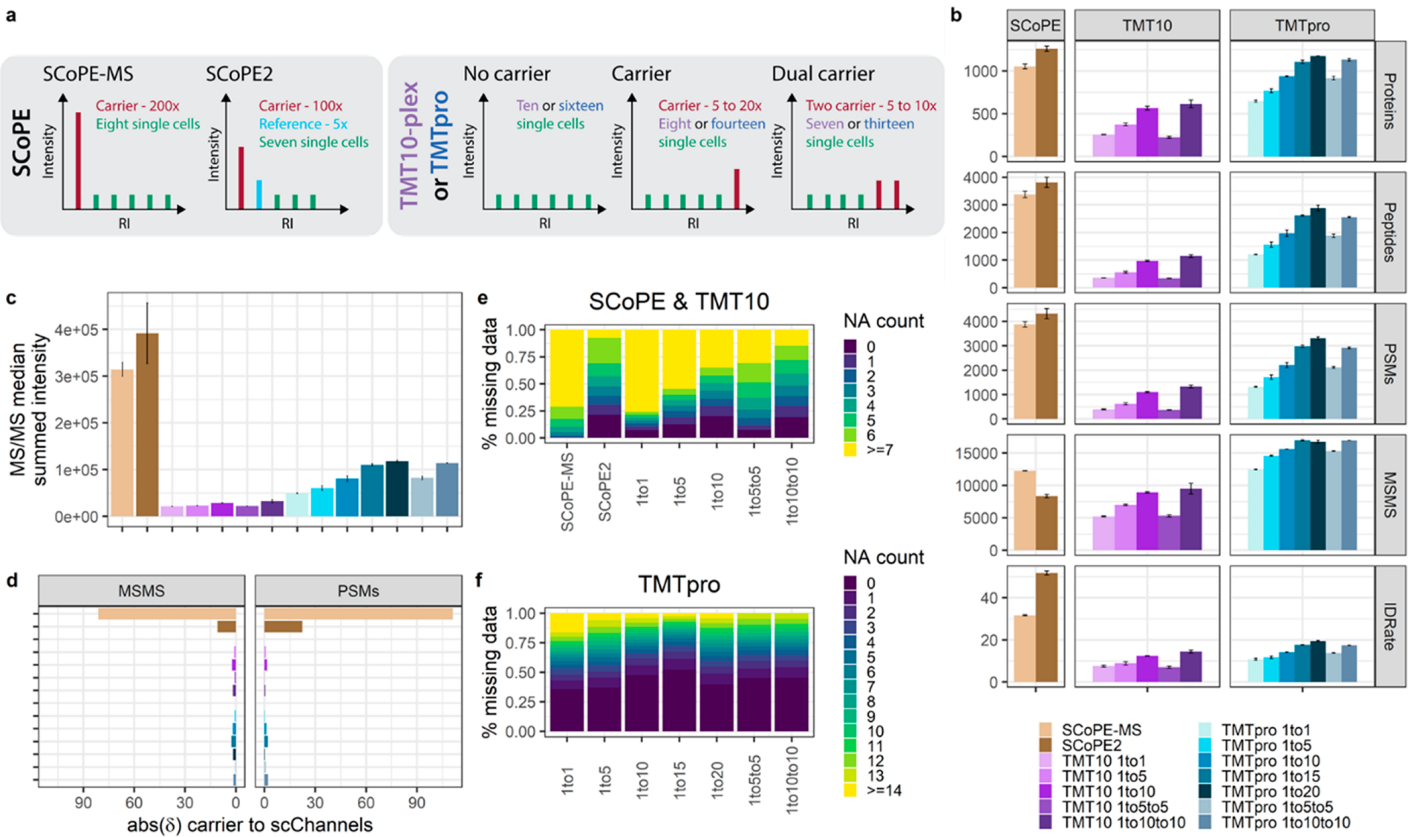
Characterization of TMT multiplexed carrier titrations. (a) Graphical illustration of experimental setups and carrier compositions. (b) Identified proteins, peptide groups, PSMs, number of MS/MS scans, and ID-rates. (c) Median summed MS/MS intensity. (d) δ-Value between expected and acquired carrier to “single-cell” ratio across all MS/MS scans or PSMs for SCoPE (brown), TMT10 (purple), and TMTpro (blue) samples at the indicated carrier spikes. Median and median absolute deviation (mad) are shown. Percent of missing quantitative data in (e) SCoPE and TMT10 and (f) TMTpro carrier titrations per PSM.

To directly compare diverse multiplexing strategies for ultralow-input samples, we performed the labeling of the HeLa digest in bulk. We combined 150 pg peptide input per TMT label, which we will from now on refer to as “single-cell”, with carrier titrations. Based on previous findings concerning accurate ratio reporting, we performed TMT10 experiments with a maximum carrier of 10×.^15^ As similar studies for the 16 channel TMTpro are lacking but several studies demonstrated the quantitative implications of a >20× carrier, we extended this titration to 20×.^9,12^ Additionally, we evaluated a “dual carrier” with the carrier distributed equally across two TMT channels to reduce the extreme ratios but still boost ions. To overcome isobaric interference of the carrier sample, we did not include adjacent channels in the quantitative evaluations. For SCoPE-MS and SCoPE2 (SCoPE) experiments, we adapted the respective acquisition parameters and experimental setup detailed in the Methods section (Figure 1a).^7,8^ SCoPE2 is described to yield about 1000 proteins from real single-cell measurements.^7^ Our reanalysis of their raw data identified up to 700 protein groups, while we reproducibly identified ∼1300 proteins from diluted bulk samples by adopting their experimental setup. Therefore, we are confident in reflecting previously published protocols accurately (Figures 1b and S1a).

### Abundant Carrier Spikes Enhance Protein Identifications but Suffer from Ratio Compression

We identified around 1000 proteins for SCoPE and TMTpro 15–20× samples. In detail, SCoPE-MS experiments with 200× carrier and 250 ms max IT yielded 30% more MS/MS scans than SCoPE2 with 100× carrier and 300 ms max. IT (Figure 1b). Nevertheless, the 50% identification rate (ID-rate) of SCoPE2 outperformed all other experimental setups presented in this study, similarly to their published raw files (i.e., 30% ID-rate) from real single-cell measurements (Figure S1a).^7^ TMTpro 10–20× samples triggered over 16 000 low-intensity MS/MS scans with only a 20% ID-rate and finally 15% fewer protein identifications compared to SCoPE2. Likewise, the 60% increased peptide amount of the respective TMTpro and TMT10 samples yields more intense MS/MS scans and superior ID-rates (Figure 1b). Additionally, the larger TMTpro reagents (419 Da) require only 30% NCE compared to 34% for the TMT10 tag (344 Da). Surprisingly, although we injected similar peptide amounts, average MS/MS scans and ID-rates declined in split-carrier experiments for TMT10 and TMTpro. We speculate that one abundant carrier relatively contributes a more productive signal and less noise compared to the two lower ones. We concluded that extreme carrier spikes result in high-intensity MS/MS spectra due to the increased peptide amount per injection but only in conjunction with increased AGC targets, and maximum IT serves the necessary fragment ions for enhanced protein identifications (Figure 1b and c).

To investigate the quantitative accuracy of multiplexed measurements, we first included all MS/MS scans with RI signals and determined the δ of expected to acquired RI intensities. Strikingly, we observed that the “single-cell” signal in SCoPE-MS experiments was compressed by close to 50%, while SCoPE2 drastically improved the ratio compression to only 10% (Figure 1d). Similarly, in published SCoPE data sets we observed severe signal compression for SCoPE-MS and only to a lesser extent in SCoPE2 (Figure S1b). Next, we only considered identified scans and observed that the most abundant MS/MS scans provide sufficient fragment ions for identification but exhibit strong ratio compression. Further, only ∼50% of all MS/MS were within the carrier range (± 50% of expected ratio) for SCoPE experiments (Figures 1d and S2). This is in stark contrast to most balanced TMT10 and TMTpro experiments with more than 80% of all MS/MS scans within the expected carrier ratio (Figure S2). We speculate that high-intensity MS/MS spectra exhibit elevated noise levels, which impact measurement accuracy in the presence of an abundant carrier and are aggravated in real single-cell samples.

**Figure 2:**
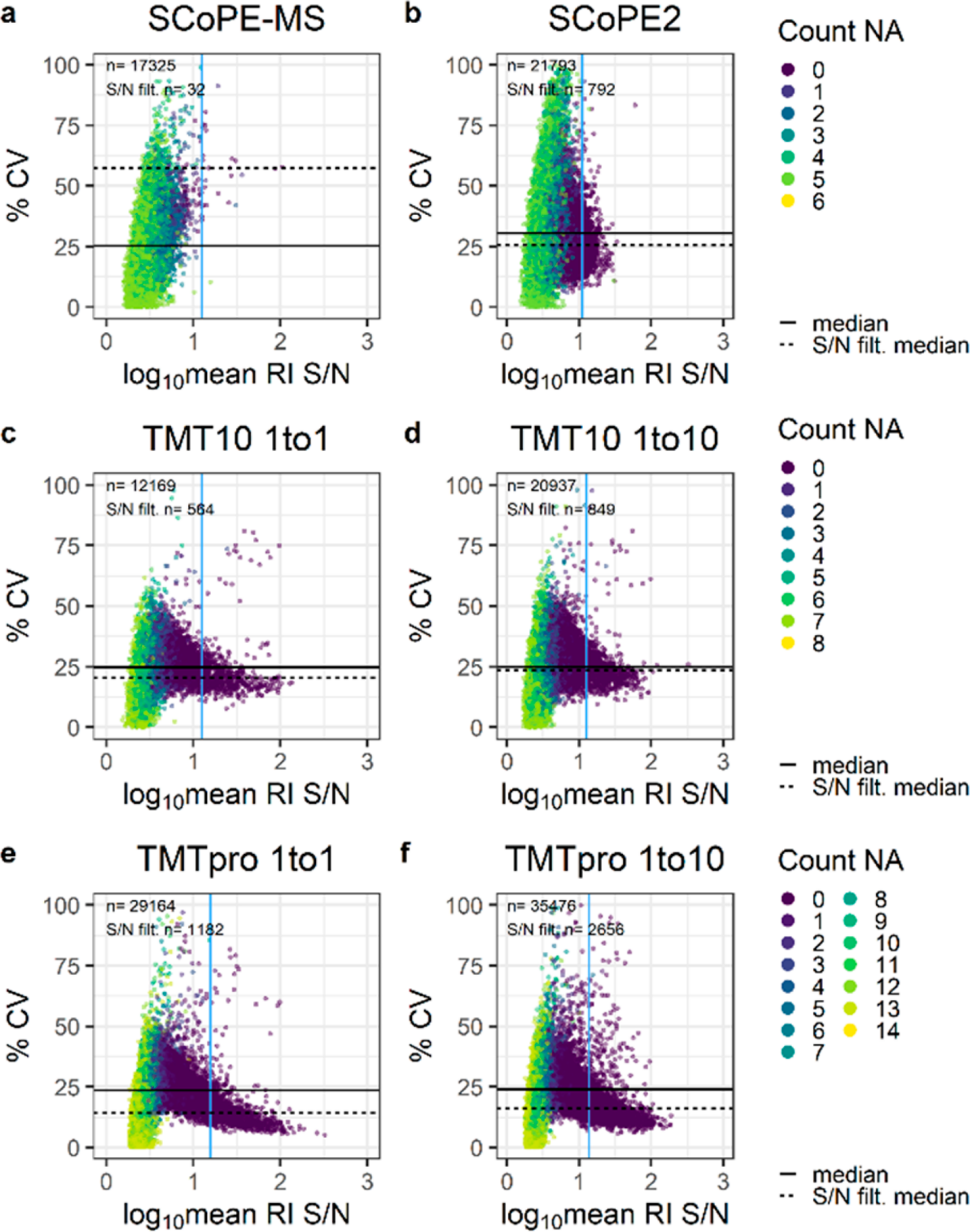
Quantification accuracy of various carrier titrations. Percent CV across “single-cell” channels and log_10_ mean RI S/N for (a and b) SCoPE, (c and d) TMT10, and (e and f) TMTpro samples at the given carrier ratios. The horizontal solid and dashed lines indicate the median S/N across all MS/MS scans and post-S/N filtering, respectively. The vertical blue line specifies the S/N filter cutoff. Colors reflect the number of missing “single-cell” RIs per MS/MS scan.

Considering the abundant carrier spike within the RI cluster, we dissected intensity profiles of individual channels and observed isobaric interference in SCoPE experiments, as expected (Figures S3a and b and S4a and b). Therefore, they exclude the adjacent channel for single cells but establish an empty or reference channel for quality control and normalization.^7,8,13^ Lower carrier ratios did not exhibit isobaric interference, but adjacent channels were nevertheless excluded from subsequent analysis (Figure S3c–f). To investigate whether measurement variation and signal intensity are parallel, we correlated the coefficient of variation (CV) between “single-cell” RIs within one MS/MS scan to the average RI S/N. For this, we combined three technical replicates, removed all MS/MS scans with only carrier or missing RIs, and determined the % CV. In our experimental setup, all “single-cell” channels have equimolar distributions, theoretically resulting in 0% CV. As expected, most MS/MS scans with low S/N and multiple missing quantitative values show high variance. Despite the enhanced average MS/MS intensity in SCoPE experiments, the mean “single-cell” S/N is lower than those for balanced TMT10 and TMTpro experiments (Figure 2a–f). While SCoPE-MS experiments present a decreased median of 25% CV compared to 30% in SCoPE2, the latter indicates a trend toward small % CV values in high S/N MS/MS scans (Figure 2a and b). Further, the 200× carrier in SCoPE-MS leads to a detrimental suppression of the “single-cell” RIs, giving rise to almost 75% missing data. In contrast, the reduced 100× carrier yielded higher “single-cell” S/N, resulting in over 90% or 75% of all MS/MS scans with at least one or two RIs, respectively (Figure 1e). This, however, disagrees with the reanalysis of published single-cell data, where SCoPE-MS presents a 4× higher single-cell RI S/N compared to SCoPE2 with almost no missing quantitative data across all PSMs (Figure S4c–e).

Based on analogous observations, quality control via RI S/N filtering was introduced with *SCPCompanion*,^12^ which we applied to our data sets to reduce the number of MS/MS scans and remove almost all scans with missing RIs. In detail, a minimum RI S/N of 12.6 for SCoPE2 eliminated over 96% of all MS/MS scans but improved the median CV by 5%. Similarly, over 90% of all MS/MS scans were removed for TMTpro no-carrier and 10× samples, but the median CV was enhanced by 10%. In most experimental setups but especially across the limited carrier TMTpro samples, high RI S/N MS/MS scans trend toward low % CV values (Figure 2a–f). This was not observed in the reanalysis of published SCoPE data sets, which we attribute mainly to biological and technical variance. Nevertheless, it is noteworthy that the quantitative confidence of real single-cell proteomics samples benefits from RI S/N filtering (Figure S4c and d). While the identifications and measurement stability of bulk diluted TMTpro >10× and SCoPE2 experiments are comparable, ratio compression and quantitative inaccuracy in the latter suggests limiting the carrier to a maximum of 20× in combination with appropriate S/N filters (Figures 1b and d, 2b–f, S2, and S3b–f).

### An Alternative Labeling Strategy Reveals Frequent Precursor Coisolation

Based on these findings, we aimed to preserve the advantages of an abundant carrier but remove the extreme ratio from the RI cluster for improved quantification accuracy. For this, in anticipation that the targeted quantitation of only “single-cell” derived peptides would greatly reduce the impact of interchannel ratio compression, we made use of the defined mass difference provided by differential labeling of the carrier and sample peptides with TMT_zero_ (224.152 amu) and TMT10 (229.162 amu) reagents, respectively. We digested the samples with Lys-C to label peptides on the C- and the N-termini, increasing the mass difference between the carrier and the “single-cell” channels.^24^ We combined the TMT10-labeled “single-cell” peptide input with an abundant TMT_zero_ carrier at varying ratios, starting with an equivalent of ten TMT10-labeled cells (i.e., 1:1) up to 200× the combined “single-cell” peptide input (i.e., 1:20; Figure 3a). Emanating from the mass separation, TMT_zero_-labeled carrier precursors highlight TMT10-labeled ions with identical characteristics. Therefore, “single-cell” precursors are selected for fragmentation despite being close to or below the detection limit, theoretically without impairing “single-cell” quantification.

**Figure 3:**
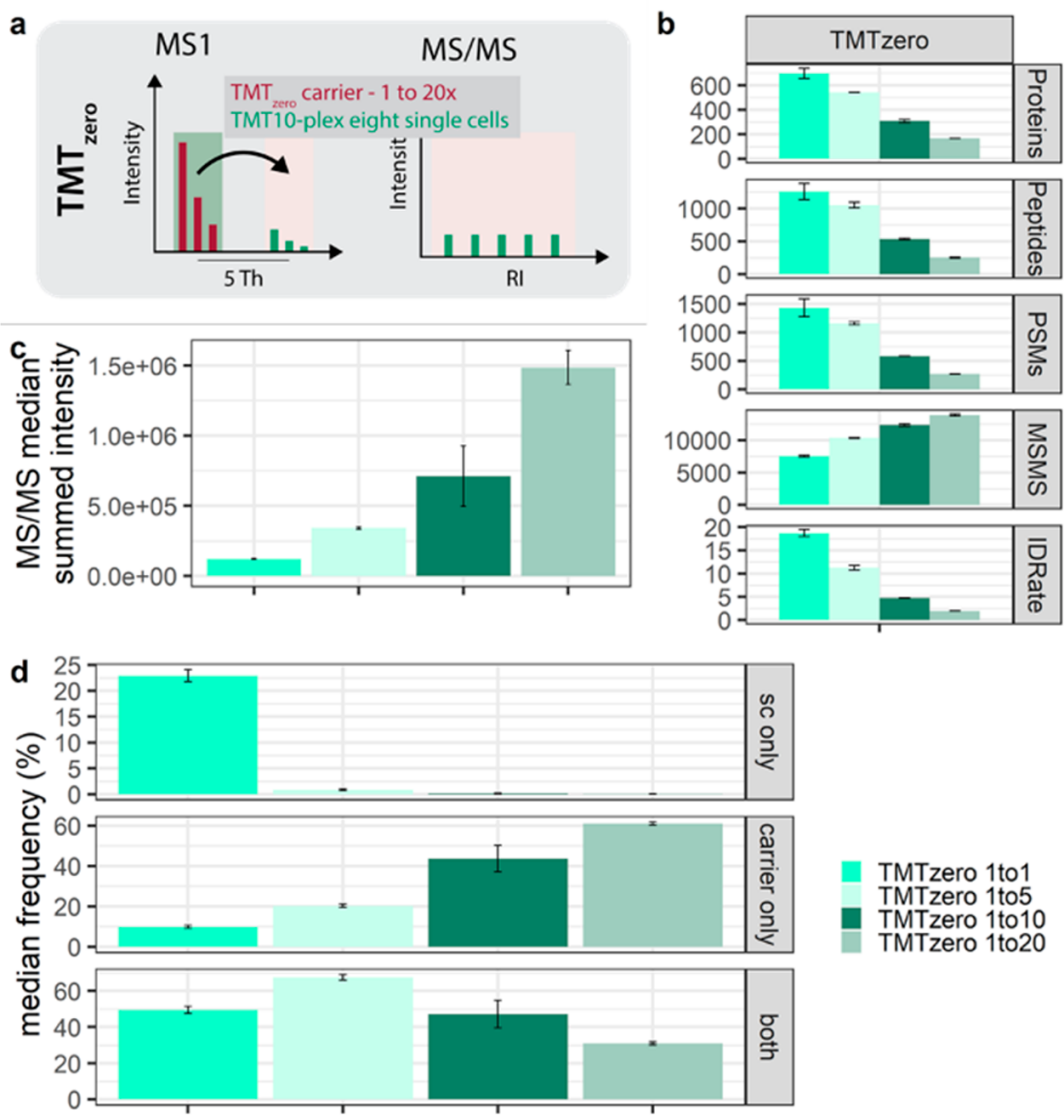
Measurement variability with intercarrier spikes using TMT_zero_. (a) Graphical illustration of the TMT_zero_ triggering strategy. (b) Protein groups, peptide groups, PSMs, MS/MS scans, and ID rates. (c) Median summed MS/MS intensity. (d) Percent median frequency of MS/MS scans across “single-cell” RIs (sc only), only carrier RI (carrier only), and coisolation of carrier and “single-cell” precursors within one MS/MS scan (both) at indicated carrier spikes. Median and mad are shown.

Like interchannel experiments, an abundant TMT_zero_ carrier repeatedly increased high-intensity MS/MS scans; however, protein identifications declined with elevated carrier ratios (Figure 3b and c, respectively). Within the TMT_zero_ approach, discrimination of “single-cell” versus carrier identifications is feasible, stemming from the different masses of the TMT10 and the TMT_zero_ tags. The 126 channel (i.e., the fragment mass of TMT_zero_) was therefore excluded to overcome the isobaric interference of mixed spectra and allow the estimation of the coisolation of a carrier precursor by the presence of a RI signal with 126.128 Da. This enables to estimate the frequency of only “single-cell”, carrier, or mixed MS/MS scans across the carrier titration. Interestingly, the relative frequency of coisolating “single-cell” and carrier precursors increases with a 5× carrier ratio but decreases at ≥10× carrier ratios (Figure 3d). Based on this, we speculate that the 20% reduced ID-rate across increasing TMT_zero_ carrier is partly due to the reduced number of TMT10 precursors, owing to the mass-based segregation of carrier and the sample peptide ion species (Figure 3b). Further, we observed that extreme congruent carrier spikes increase the chance of isolating only a carrier precursor fivefold compared to balanced experiments (Figure 3d). These findings indicate that, in conjunction with an abundant carrier, it is likely that most PSMs correspond to a carrier-derived identification rather than single cells. Consequently, the carrier must equally represent all single-cell precursors for accurate acquisition, which could be challenging for heterogeneous samples.

Despite low protein identifications, we evaluated the measurement stability and quantification accuracy of the TMT_zero_ experimental setups. Corroborating earlier observations with similar interchannel carrier ratios, we observed a stable “single-cell” signal but elevated isobaric interference in the 126 and adjacent channels (Figure S5a–c). While the median CV below 25% across all MS/MS scans in TMT_zero_ 1× experiments decreased to only 13% after S/N filtering, a ≥10× carrier spike resulted in frequent missing values and up to 30% CV (Figure S5d–f). We conclude that removing the carrier from the multiplexed “single cells” via TMT_zero_ elevates “single-cell” RI S/N but at extreme ratios that impair protein identifications and measurement accuracy (Figure 3b and Figure S5d–f). Moreover, the mass difference between “single cells” and the carrier revealed close to 50% coisolation and up to 60% carrier-only quantitative data in imbalanced ultralow input samples (Figure 3d). This suggests that a congruent carrier indeed improves MS/MS triggering and serves fragment ions; however, the identical features do not discern between solely a carrier or a “single-cell” PSM. Consequently, we speculate that all multiplexed ultralow-input experiments suffer similar frequencies of coisolation and convoluted RI clusters.

### Intentional Coisolation Reduces Missing Data in Ultralow-Input Samples

**Figure 4:**
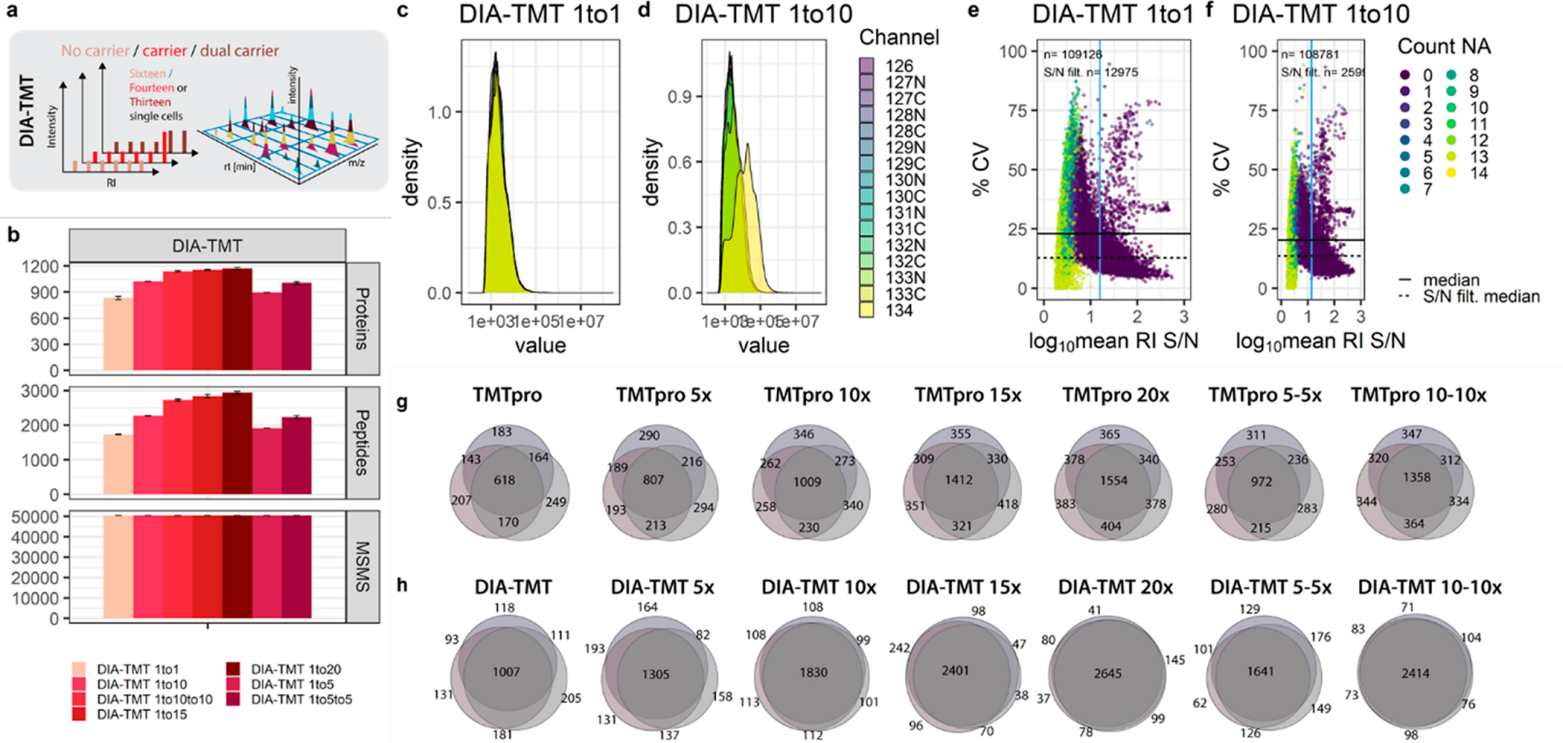
Impact of intentional coisolation on “single-cell” variability and accuracy. (a) Graphical illustration of DIA-TMT acquisition strategies with carrier titrations. (b) Protein groups, peptide groups, and MS/MS scans of DIA-TMT samples at indicated carrier spikes. Median and mad are shown. (c and d) RI intensity distributions across all MS/MS scans and (e and f) %CV against log_10_ mean RI S/N for DIA-TMT samples at indicated carrier spikes. The horizontal solid and dashed lines indicate the median S/N across all MS/MS scans and post-S/N filtering, respectively. The vertical blue line indicates the S/N filter cutoff. Colors indicate the number of missing single-cell RIs. Replicate overlap of (g) DDA and the corresponding (h) DIA-TMT samples based on unique peptides.

TMT_zero_ experiments allowed us to estimate unintentional coisolation, RI convolution, and its impact on MS/MS-based quantification accuracy (Figures 3c and d and S5a–f), as previously discussed by many.^12,16,24−30^ Additionally, we and others found detrimental amounts of missing data in multibatch data-dependent proteomics experiments (Figure 1e and f).^20,23,31−33^ This is most prominently addressed via data-independent acquisition (DIA), which our group recently extended to multiplexed samples.^23^ While coisolation is non-negotiable with our 5 Th DIA-TMT method (i.e., in contrast to 0.7 Th in standard DDA), the prescheduled acquisition strategy theoretically generates no missing data across multiple analytical runs (Figure 4a). In detail, our small-window DIA-TMT method allows us to uniformly generate abstract 3D maps comprised of RT, precursor *m*/*z*, and RI intensity. These 3D maps or “proteome signatures” entail convoluted RI quantification of a reproducible set of in bona fide precursors across all analytical runs. While we intentionally coisolate multiple precursors to expedite sampling and provide consistent “proteome signatures”, convoluted RIs distinguish cell types down to single protein knockouts.^23^ In contrast, the stochastic nature of data-dependent acquisition (DDA) methods, especially in analyzing ultralow-input samples, generates detrimental amounts of missing data. Consequently, this requires most quantitative data to be computationally generated across large sample cohorts. However, the obvious application of any single-cell technology to characterize tissues or cellular subpopulations requires quantitative profiles of hundreds or thousands of samples, which are facilitated via our sensitive DIA-TMT strategy.^23^

Accordingly, we evaluated the quantification accuracy and reproducibility of DIA-TMT in conjunction with the TMTpro carrier titrations. Interestingly, the DIA-TMT samples yielded slightly higher protein identifications at PSMs similar to those of corresponding DDA measurements (Figure 1b and 4b). We speculate that this is because of the decreased cycle time and optimized fragmentation due to the stepped collision energy providing optimal fragmentation for coisolated precursors with different charge states. Further, the measurement variance of no-carrier samples is comparable to that of DDA experiments but increases in combination with a congruent carrier spike (Figures 4c and d and Figure S3e). While we observed similar overall RI intensities for DDA and DIA measurements, the median RI S/N increased by 40% for the latter (Figures 2 e and f and 4 e and f).

Interestingly, despite some distorted scans at high RI S/N, the DIA-TMT strategy presented close to 20% CV between “single cells” across all carrier titrations. As previously discussed, S/N filtering further decreased the median CV to around 10%, corresponding to the lowest “single-cell” variation across all experiments (Figure 4e and f). However, to constitute the complete “proteome signature”, the convoluted RI cluster attributes were quantified to a set of coisolated precursors rather than a single peptide species. Lastly, to directly compare replicate overlaps in DDA and DIA, we intersected unique peptide identifications. DIA-TMT increased replicate overlap by 25% in contrast to corresponding DDA samples across all carrier titrations (Figure 4g and h). With low measurement variance, exceptional accuracy, and close to 90% replicate overlap, DIA-TMT demonstrates its potential to overcome missing data at comparable quantitative accuracy in ultralow input samples.

## Conclusion

**Figure 5:**
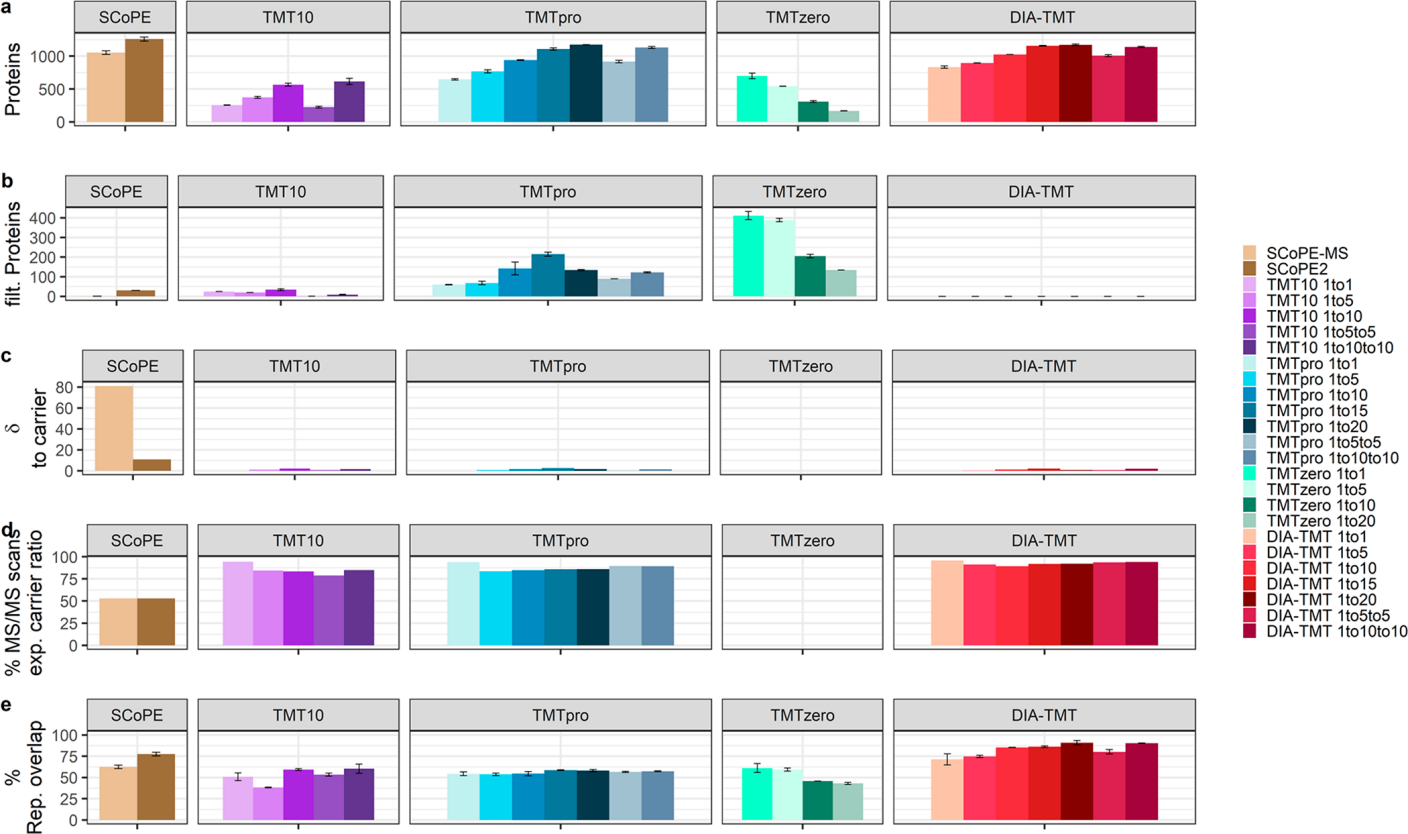
Cumulative comparison of measurement accuracy, variance, and reproducibility across all experimental setups. (a) Identified proteins groups; (b) S/N-filtered protein groups (of note, the DIA-TMTpro panel is not included as single MS/MS scans do not give rise to protein identifications); (c) δ-carrier to “single-cell” intensities (optimal value of 0); (d) percent of MS/MS scans with “single-cell” RI within ±50% of expected carrier ratio (of note, TMT_zero_ is not included for panels c and d as the “single-cell” carrier ratio cannot be determined within one MS/MS scan); and (e) percent replicate overlap across triplicates based on unique peptides for SCoPE (brown), TMT10 (purple), TMTpro (blue), TMT_zero_ (turquoise), and DIA-TMT (red). Bar graphs display the median, and error bars indicate the mad.

We dissect different multiplexing strategies at extensive carrier titrations to investigate the impact on ID-rates, reproducibility, quantification accuracy, and measurement interference. Interestingly, we observe almost a linear increase in protein identifications across the low carrier titrations for both isobaric reagents. Moreover, we find that congruent carrier spikes effectively contribute ions to the “single cells” and consequently increase MS/MS intensities. Already, a small carrier (<20×) improves ID-rates, which eventually plateau at ≥100× ratio for 60 min gradients. The SCoPE2 acquisition parameters and the 50% carrier decrease compared to that of SCoPE-MS reduced ion suppression, increased ID-rates, and improved measurement accuracy (Figures 1b and d, 2a and b, 5a–d, and S2). While even lower carrier spikes on average resulted in less intense MS/MS scans and lower ID-rates, with less extreme RI ratios, we observed no ratio compression, less measurement variability, and predominantly fewer missing values (Figures 1b–f, 2c–f. and 5a–d). However, even nonstringent S/N filtering often eliminated around 90% of MS/MS scans, suggesting that the RI S/N of such ultralow input samples is suboptimal across experimental setups (Figure 2c–f). This is especially concerning as the diluted bulk digests utilized in this study contain less chemical background than real single-cell samples.^12,34^ As expected, *SCPCompanion*-advised RI S/N filtering prior to database searching dramatically reduced protein identifications across all conditions and highlighted the carrier limitation of <20× (Figure 5b).^12^ Interestingly, the carrier abundance in TMT_zero_ samples parallels with the frequency of carrier coisolation and therefore decreasing protein identifications. Despite that, 50% more protein groups surpassed RI S/N filtering for TMT_zero_ compared to all other experimental setups (Figure 5a and b). We therefore speculate that our current TMT_zero_ acquisition strategy, despite being highly accurate and selective, suffers from inefficient triggering, which could be improved with more stringent precursor selection. Further, the quantitative accuracy of S/N-filtered MS/MS scans indicates that a real-time search MS3-based approach to offset-trigger solely if the carrier precursor is identified would benefit the method (Figure S5d–f).^35,36^

Moreover, the no-carrier samples demonstrated outstanding measurement accuracy and reduced variability, especially in TMTpro samples (Figures 2c and e, 5c and d, and S3c and e). Despite a comparable peptide input and a total ion current of the 10× TMT10 or the 5× TMTpro samples, the latter yielded 25% more MS/MS scans and protein identifications. Due to similar “single-cell” CVs with both isobaric reagents, we speculate that the global identification increase with TMTpro results from different fragmentation patterns (Figure 1b). This further provided a major advantage to overcome detrimental precursor stochasticity and improve reproducibility with our multiplexed DIA strategy. Consequentially, the DIA-TMT acquisition of TMTpro samples provided comparable protein identifications and superior data completeness, resulting in close to 100% replicate overlap at reduced measurement variance (Figures 4b–h and 5a–e).

The alternative triggering via TMT_zero_ confirmed that abundant carrier spikes dominate low-abundance “single-cell” MS/MS spectra, even if segregated from the RI cluster (Figure 3c and d). Importantly, such coisolated MS/MS spectra may be comprised mainly of carrier *b*- and *y*-ions, while the presence of any RI signal is used for quantification of “single cells”.^12,13^ Even though high TMT_zero_ RI intensities indicate that the acquisition strategy might overestimate the prevalence of mixed spectra, already at ≥5× carrier spikes most MS/MS scans either comprise only carrier or coisolated precursors (Figures 3d and S5a–c). While background and possible contaminations are reduced to a minimum in our bulk dilutions, this might affect the biological interpretation of real single-cell samples. Contrarily, the intentional coisolation in a prescheduled acquisition scheme using DIA-TMT successfully defines cell-types and underrepresented single protein knockouts and presents less quantitative variance at theoretically no missing data.^23^ The need for quantitative data imputation in standard single-cell DDA data is highly elevated compared to standard input, while the absence of technical replicates challenges the reliability.^11,20,37−39^ Despite the intentional coisolation in DIA-TMT, eliminating precursor stochasticity drastically improved both the accuracy and the sensitivity (Figure 4b–f).^23^ Hence, we speculate that large proportions of computationally generated quantitative data introduced by reduced replicate overlap in combination with precursor coisolation and extreme carrier ratios are particularly error prone.

We present a comprehensive overview of currently available multiplexed single-cell proteomics setups considering protein identifications, measurement variance, quantitative accuracy, and missing data. We find that specific experimental questions require individual prioritization of parameters when designing ultralow-input or single-cell studies. Based on these findings, we conclude that limiting carrier spikes (i.e., ≤20×) is pivotal for accurate single-cell proteomics analysis and thus any biological interpretation (Figure 5a–e). With more sensitive instrumentation and dedicated experimental approaches, single-cell proteomics has achieved remarkable proteome depth and throughput. Nevertheless, many parameters such as the cell state, sample preparation, chromatography, and ultimately the acquisition style impact data quality. We are confident that efficient sample preparation workflows, novel instrumentation, and tightly controlled computational approaches will drive biological applications and further demonstrate the impact of hypothesis-free proteome measurements at a single-cell resolution.

## Supporting Information Available

Additional experimental details, including in-depth ratio suppression and measurement variance for all conditions contained in the manuscript, a comparative reanalysis of the published SCoPE data sets, and measurement accuracy of TMT_zero_ (PDF)

## Notes

All mass spectrometry-based proteomics data have been deposited to the ProteomeXchange Consortium via the PRIDE partner repository with the data set identifier [PXD027912]. Reviewer account details are as follows: username reviewer_pxd027912@ebi.ac.uk, password G1jNd1XX.
