# Supplementary material for: Quantitative Accuracy and Precision in Multiplexed Single-Cell Proteomics
Source: Anal Chem. 2021 Dec 30;94(5):2434–43. doi: 10.1021/acs.analchem.1c04174 (PMC8829824; doi:10.1021/acs.analchem.1c04174)
Supplement: Supplementary file 1 — ac1c04174_si_001.pdf [file ac1c04174_si_001.pdf]

Supporting Information

Quantitative accuracy and precision in multiplexed single-cell proteomics.

Authors:

Claudia Ctortecka[1,*], Karel Stejskal[1,2,3], Gabriela Krššáková[1,2,3], Sasha Mendjan[2] and Karl Mechtler[1,2,3,*].

Affiliations:

1 Research Institute of Molecular Pathology (IMP), Vienna BioCenter (VBC), Campus-Vienna-Biocenter 1, 1030 Vienna, Austria.

2 Institute of Molecular Biotechnology of the Austrian Academy of Sciences (IMBA), Vienna BioCenter (VBC), Dr. Bohr-Gasse 3, 1030 Vienna, Austria.

3 The Gregor Mendel Institute of Molecular Plant Biology of the Austrian Academy of Sciences (GMI), Vienna BioCenter (VBC), Dr. Bohr-Gasse 3, 1030 Vienna, Austria.

* Correspondence:

Claudia Ctortecka

Claudia.ctortecka@imp.ac.at

Karl Mechtler

Karl.mechtler@imp.ac.at

Table of Content:

Figure S1: *Re-analysis of public SCoPE data.*

Figure S2: *Ratio compression for SCoPE, TMT10-plex and TMTpro samples at various carrier spikes.*

Figure S3: *Measurement stability, RI variability and quantification accuracy at various carrier ratios.*

Figure S4: *Measurement stability and variance of public SCoPE data.*

Figure S5: *Measurement stability and accuracy of TMT_{zero} experiments.*

Figure S6: *Ratio compression in DIA-TMT experiments.*

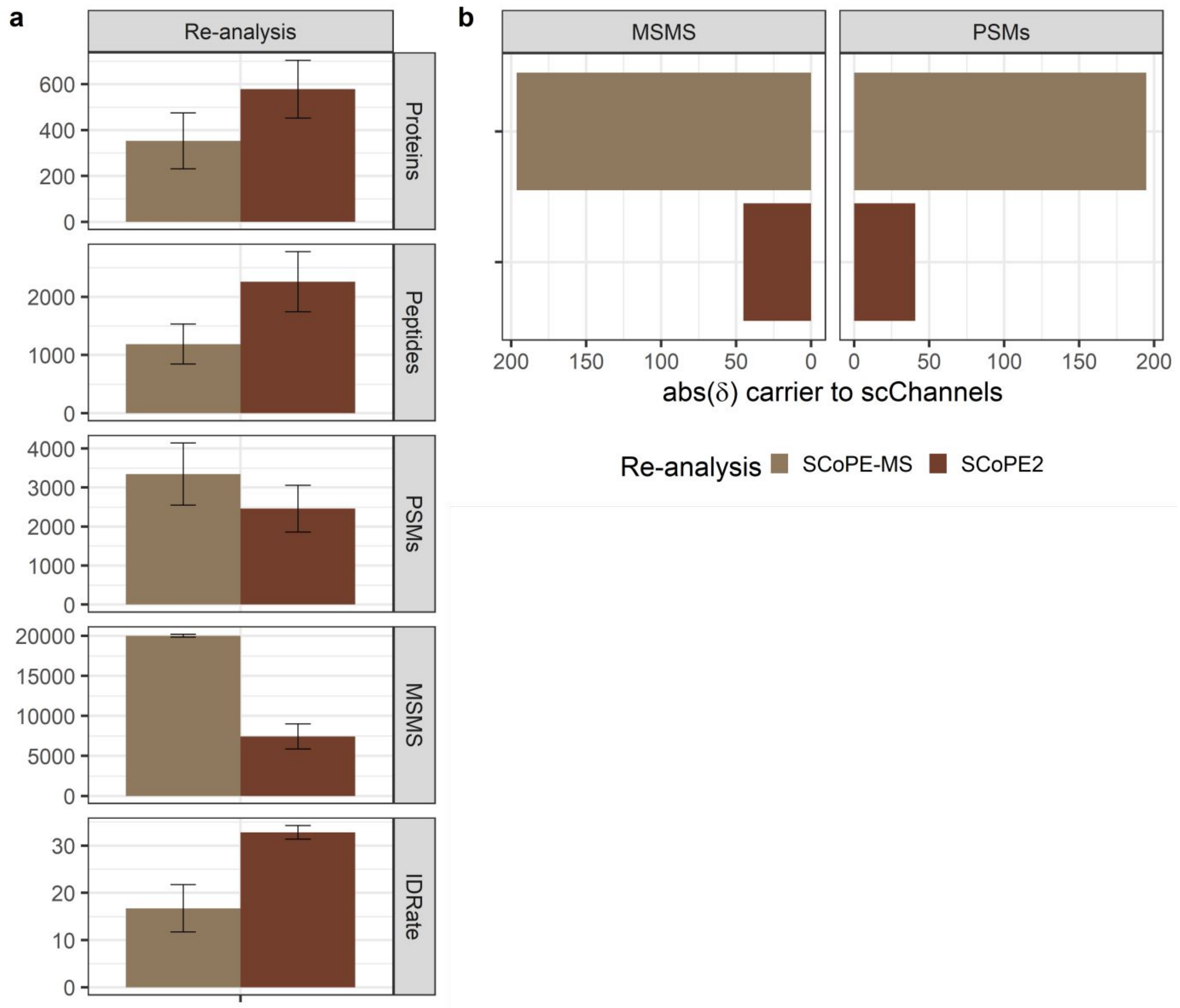

Figure S1: Re-analysis of public single cell data, **(a)** Identified proteins, peptide groups, PSMs, number of MS/MS scans, ID-rates and **(b)** delta between expected and acquired carrier to single cell ratio across all MS/MS scans or PSMs of published SCoPE-MS (light brown) and SCoPE2 (dark brown). Median and median absolute deviation (mad) is shown.

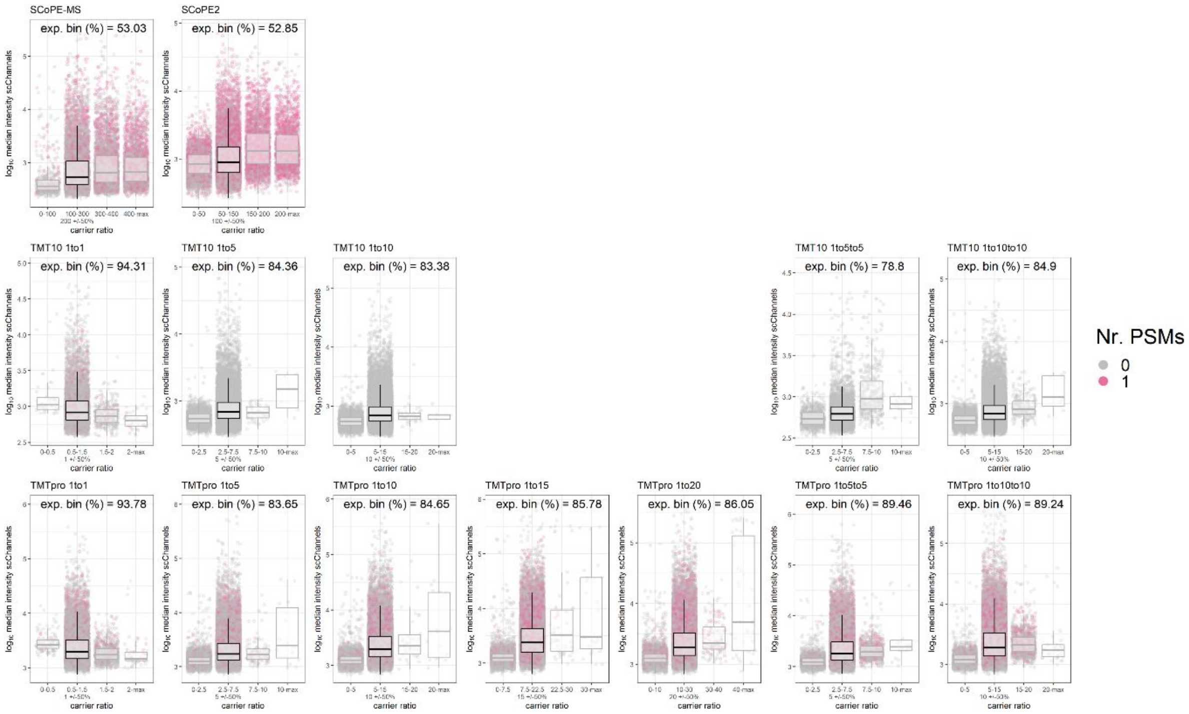

Figure S2: Ratio compression for SCoPE, TMT10-plex and TMTpro samples at various carrier spikes. Log_{10} median RI intensity of all MS/MS scans and binned ratios between ‘single cells’ and the carrier is displayed. Identified and unidentified MS/MS scans are indicated in grey or pink, respectively. The expected bin (carrier +/- 50 %) is highlighted in black, and the percent of MS/MS scans within those is indicated.

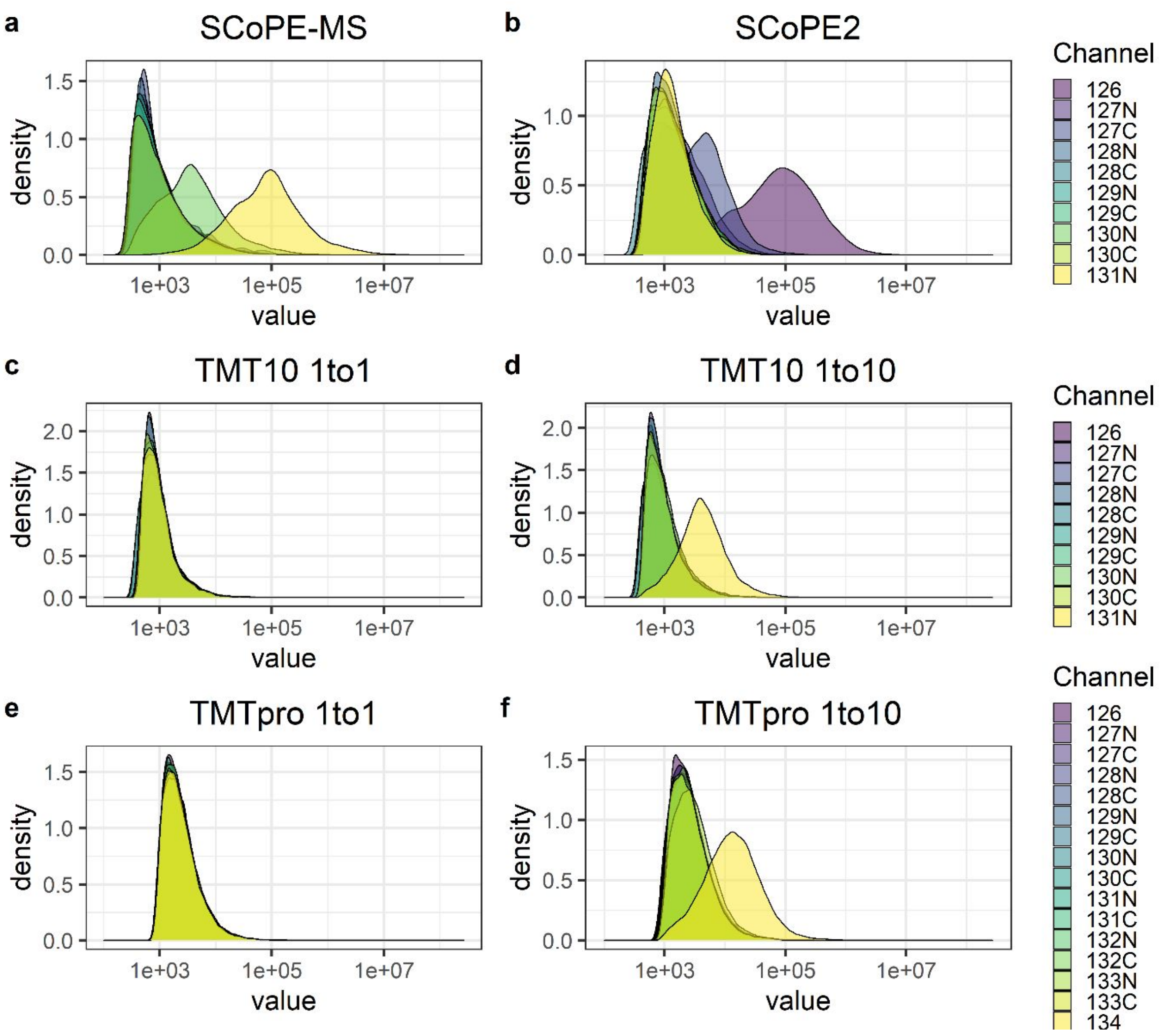

Figure S3: Measurement stability and RI variability at various carrier ratios. RI intensity distributions based on all MS/MS scans for **(a-b)** SCoPE, **(c-d)** TMT10-plex and **(e-f)** TMTpro experiments at indicated carrier spikes.

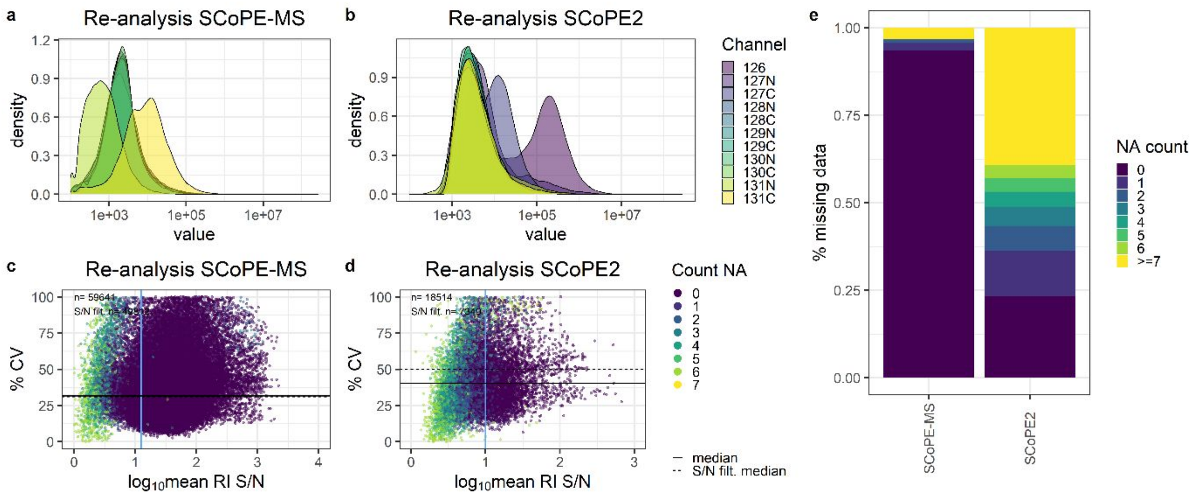

Figure S4: Measurement stability and variance of public SCoPE-MS and SCoPE2 data. **(a-b)** RI intensity distributions based on all MS/MS scans and **(c-d)** percent CV across 'single cell' channels and $\log_{10}$ mean RI S/N for publicly available SCoPE data. The horizontal solid line and dashed lines indicate median S/N across all MS/MS scans or post-S/N filtering, respectively. The vertical blue line specifies the S/N filter cut-off. Colors reflect the number of missing 'single cell' RIs per MS/MS scan. **(e)** Percent missing quantitative data in publicly available SCoPE data per PSM.

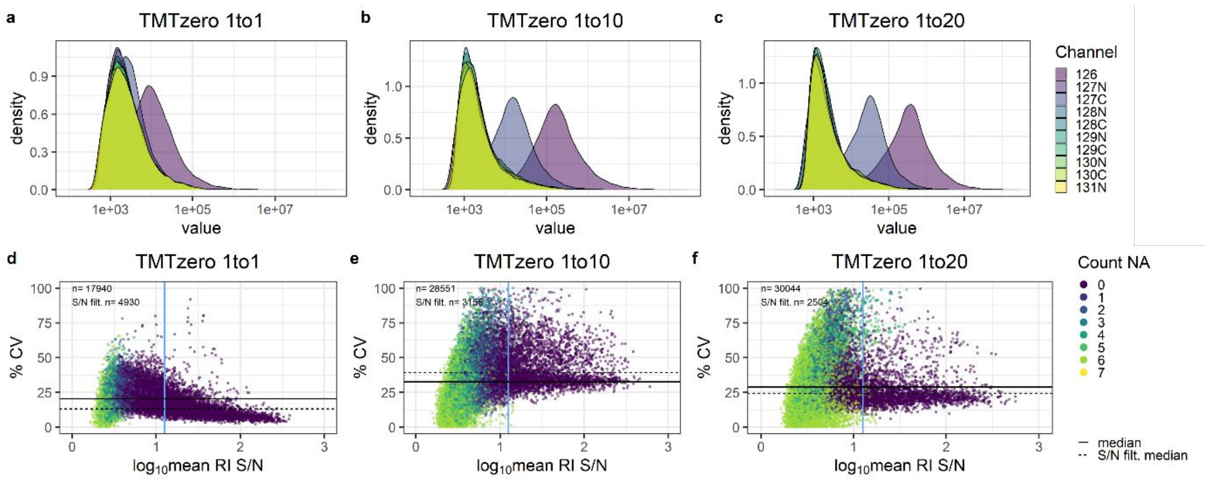

Figure S5: Measurement accuracy of TMT_{zero} experiments. **(a-c)** RI intensity distribution across all MS/MS scans in TMT_{zero} experiments. Colors indicate different TMT channels. **(d-f)** Median percent CV and $\log_{10}$ mean RI S/N at indicated TMT_{zero} carrier spikes. Horizontal solid and dashed lines indicate median S/N across all MS/MS scans or post-S/N filtering, respectively. The vertical blue line indicates the S/N filter cut-off. Colors indicate the number of missing ‘single cell’ RIs per MS/MS scan.

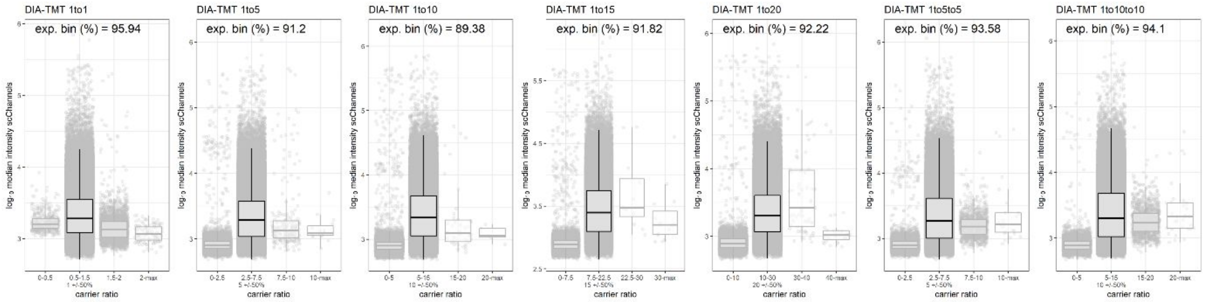

Figure S6: Ratio compression in DIA-TMT experiments. Log_{10} median RI intensity across all MS/MS scans is displayed with binned ‘single cell’ to carrier channels ratios. The expected ratio (carrier +/- 50%) is highlighted in black, and percent of MS/MS scans within this bin is indicated.
